# Nanobodies as modulators of inflammation: potential applications for acute brain injury

**DOI:** 10.3389/fncel.2014.00344

**Published:** 2014-10-21

**Authors:** Björn Rissiek, Friedrich Koch-Nolte, Tim Magnus

**Affiliations:** ^1^Department of Neurology, University Medical Center Hamburg-EppendorfHamburg, Germany; ^2^Department of Immunology, University Medical Center Hamburg-EppendorfHamburg, Germany

**Keywords:** nanobodies, single domain antibodies, VHH, blood-brain barrier, neuroinflammation

## Abstract

Nanobodies are single domain antibodies derived from llama heavy-chain only antibodies (HCAbs). They represent a new generation of biologicals with unique properties: nanobodies show excellent tissue distribution, high temperature and pH stability, are easy to produce recombinantly and can readily be converted into different formats such as Fc-fusion proteins or hetero-dimers. Moreover, nanobodies have the unique ability to bind molecular clefts, such as the active site of enzymes, thereby interfering with the function of the target protein. Over the last decade, numerous nanobodies have been developed against proteins involved in inflammation with the aim to modulate their immune functions. Here, we give an overview about recently developed nanobodies that target immunological pathways linked to neuroinflammation. Furthermore, we highlight strategies to modify nanobodies so that they can overcome the blood brain barrier and serve as highly specific therapeutics for acute inflammatory brain injury.

## From llama heavy-chain-only antibodies to single domain nanobodies

Mammalian immunoglobulins are composed of two heavy and two light chains which together form the antigen-binding paratope. In 1993, the group of Raymond Hamers demonstrated the existence of a new type of immunoglobulin in the serum of camels (*Camelus dromedarius*). These antibodies consist of heavy-chain dimers devoid of light chains, which brought them the name “heavy-chain-only antibodies” (HCAbs; Hamers-Casterman et al., 1993). These HCAbs are present in all members of the camelid family and account for 30 to 75% of circulating immunoglobulins (Hamers-Casterman et al., 1993; Sundberg and Mariuzza, 2002; Blanc et al., 2009; Muyldermans, 2013).

**Figure 1:**
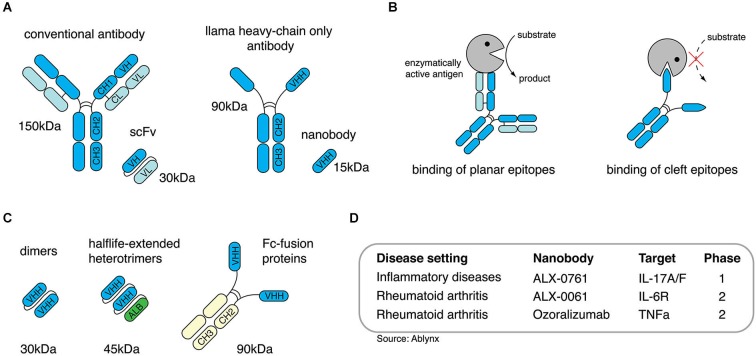
**Nanobodies are single-domain antibodies derived from llama heavy-chain-only antibodies. (A)** Conventional antibodies from mammals are composed of two heavy- and two light chains, camelidae additionally express antibodies devoid of ligh chains, so called heavy-chain only antibodies. **(B)** Heavy-chain-only antibodies can bind to molecular crevices thereby blocking the active site of enzymes. **(C)** Nanobodies derived from heavy-chain-only antibodies can be engineered as dimers, half-life-extended heterotrimers containing an anti-albumin nanobody or as dimeric Fc-fusionprotein.** (D)** Anti-inflammatory nanobodies are currently evaluated in clinical trials.

Structurally, the heavy-chains of HCAbs are composed of the antigen-binding variable domain (VHH) followed by a hinge region and two constant domains (CH2 and CH3), whereas the CH1 domain known from conventional antibodies is missing (Figure 1A; Hamers-Casterman et al., 1993). Apart from their unusual architecture, HCAbs also differ from conventional antibodies in their antigen recognition: VH and VL of conventional antibodies usually form a concave or flat shaped paratope suited for the binding of small molecules, peptides, or large antigens (Sundberg and Mariuzza, 2002; Blanc et al., 2009; Muyldermans, 2013). The paratope formed by a VHH domain, however, shows a convex shape and, therefore, enables the binding to molecular cavities or clefts, e.g., the active site of enzymes. Many enzyme-specific VHH domains thereby act as antagonists (Figure 1B). This unique feature could be attributed to the long complementarity determining region 3 (CDR3) of the VHH domain which is able to form finger-like extensions (De Genst et al., 2006).

With approximately 15 kDa VHHs are the smallest naturally occurring antigen-binding protein domains. The name “nanobodies” was coined to reflect the small size of VHHs as recombinant proteins (Muyldermans, 2013). In order to generate nanobodies from HCAbs, llamas are immunized and boosted with the desired antigen. After the last boost, B cells are collected from peripheral blood to isolate mRNA, which is transcribed into cDNA. The gene region encoding for the VHH domain can be amplified via PCR and cloned into a phagemid vector. This strategy generates phages that express one particular nanobody clone on their surface and, at the same time, carry the DNA sequence encoding for this specific nanobody. Applying the phage display technology finally allows the selection of nanobody clones against the desired antigen (Clackson et al., 1991). Taken together, this approach allows the selection of target-specific nanobodies and, simultaneosly, delivers the DNA sequence coding for the selected nanobodies which then can be further used for recombinant expression (Wesolowski et al., 2009).

Most generated nanobodies are stable at high temperatures, low and high pH, and other stringent conditions (Arbabi Ghahroudi et al., 1997; Dumoulin et al., 2002). Additionally, phage display selection can be performed under harsh conditions, e.g., the presence of detergents to improve the selection of more resistant clones. If applied *in vivo*, nanobodies display low toxicity and immunogenicity due to their small size, their relatively high sequence identity to human VH, and to their rapid clearance from the periphery via the kidney (Hamers-Casterman et al., 1993; Muyldermans, 2013). To increase the *in vivo* half-life nanobodies can be reformatted (converted into other formats by genetic engineering), e.g., to homodimers, heterotrimers containing an anti-serum-albumin nanobody (Sundberg and Mariuzza, 2002; Coppieters et al., 2006; Tijink et al., 2008; Blanc et al., 2009; Muyldermans, 2013) or nanobody-Fc-fusion proteins (Figure 1C). Thereby, nanobodies can be tailored for the desired *in vivo* application, e.g., small monomers for short-term *in vivo* imaging or half life-extended nanobodies for long-term therapeutic treatment (Hamers-Casterman et al., 1993; Hassanzadeh-Ghassabeh et al., 2013). The potential to antagonize targeted antigens, the high stability, the low toxicity and the possibility to tailor them for *in vivo* applications makes nanobodies a promising new generation of therapeutic proteins. To date, several anti-inflammatory nanobodies are in clinical trials (Figure 1D), and more than 700 persons have received nanobodies in clinical trials without any adverse off-target side effects (Van Bockstaele et al., 2009; Williams, 2013).

## Nanobodies as modulators of immune cells and inflammation

In order to fight infectious diseases, numerous nanobodies have been generated against bacterial and viral antigens to prevent or ameliorate pathogenicity (Sundberg and Mariuzza, 2002; Blanc et al., 2009; Wesolowski et al., 2009; Muyldermans, 2013). More recently, key players of immunological pathways have come into focus as targets for nanobodies in order to modulate immune responses. This has resulted in the generation of nanobodies directed against Fc-receptors (FcR), chemokine receptors, chemokines, cytokines, and ecto-enzymes. These nanobodies often show high target specificities and are able to modulate the function of their target in an agonistic or antagonistic fashion.

### Nanobodies directed against Fc-receptors

Fc receptors are expressed on the cell surface of diverse immune cells and are able to bind the Fc portion of antibodies thereby conducting either stimulatory or inhibitory signals, depending on the Fc receptor class (De Genst et al., 2006; Nimmerjahn and Ravetch, 2007). In 2008, Behar et al. described the isolation of Fc-γ-RIII-specific nanobodies from a llama immune library (Behar et al., 2008; Muyldermans, 2013). The selected nanobodies (C21 and C28) showed specific binding to both, Fc-γ-RIIIB and Fc-γ-RIIIA, and no binding to Fc-γ-RI or Fc-γ-RII. Binding of the Fc-part of an antibody to the Fc-γ-RIII on NK cells conducts an activating signal leading to the release of the proinflammatory cytokine interferon gamma (IFNγ). Binding of nanobodies C21 and C28 in an agonistic fashion to Fc-γ-RIIIA on human NK cells induced the expression of IFNγ (Clackson et al., 1991; Behar et al., 2008). In later studies, these nanobodies were used to generate Fab-like bispecific antibodies containing one nanobody directed against the Fc-γ-RIIIA and one directed against the carcinoembryogenic antigen (CEA; Behar et al., 2009; Wesolowski et al., 2009). By this strategy, the agonistic anti-Fc-γ-RIIIA nanobodies could be targeted to CEA^+^-tumor cells where they activate NK cells *in situ* inducing the lysis of the tumor cells. Further, injection of these bispecific constructs reduced the tumor growth in immunodeficient mice xenografted with CEA^+^-tumor cells when co-administered with human peripheral blood mononuclear cells (PBMCs; Arbabi Ghahroudi et al., 1997; Dumoulin et al., 2002; Rozan et al., 2013). It has to be evaluated whether FcR targeting nanobodies could also be applied as therapeutics for acute brain injury. A study using Fc-γ-R deficient mice showed a reduced infarct size compared to WT animals which could be linked to decreased microglia activation (Komine-Kobayashi et al., 2004).

### Nanobodies directed against chemokine receptors and chemokines

The generation of functional monoclonal antibodies against G-protein coupled receptors (GPCRs) such as chemokine receptors is notoriously difficult. With their unique binding features, nanobodies display a promising alternative for the generation of functional biologics to modulate chemokine receptor function, e.g., to inhibit immune cell migration to inflammatory sites. In 2010, the group of Martine Smit reported the generation of two nanobodies that specifically target the chemokine receptor CXCR4 (Jähnichen et al., 2010). Nanobodies 238D2 and 238D4 showed potent competitive inhibition of CXCL12 binding to CXCR4. When injected into monkeys, anti-CXCR4 nanobodies induced the mobilization of hematopoetic stem cells by disrupting the CXCR4/CXCL12 axis contributing to the residence of hematopoetic stem cells in the bone marrow. In 2013, the same group reported the generation of antagonistic nanobodies targeting CXCR7. Injected into mice, these nanobodies showed beneficial effects in an *in vivo* xenograft model of head and neck cancer (Maussang et al., 2013). Simultaneously, the same group published a panel of nanobodies specifically targeting CCL2, CCL5, CXCL11 and CXCL12. Binding of nanobodies to CXCL11 and CXCL12 inhibited chemokine receptor binding and thereby preventing chemokine receptor activation induced cell migration *in vitro* (Blanchetot et al., 2013). Since diverse chemokines and their receptors are known to contribute to the migration of immune cells to the brain after brain damage (Amantea et al., 2009), the nanobodies described above might be a promising therapeutic alternative for the treatment of acute brain injury.

### Nanobodies directed against cytokines

Targeting and neutralization of proinflammatory cytokines by monoclonal antibodies is a promising strategy for the treatment of inflammatory diseases (Kopf et al., 2010). Tumor necrosis factor α (TNFα) was the first cytokine functionally targeted by nanobodies (TR2 = anti-human TNFα, MT1 = anti-mouse TNFα). Expressed as bivalent molecules TR2-TR2 nanobodies showed a slightly higher neutralizing capacity than the TNFα neutralizing biologicals infliximab, adalimumab and etanercept (Coppieters et al., 2006). Further, heterotrimeric nanobodies (MT1-MT1-AR1) consisting of two MT1 and one serum albumin-binding nanobody (AR1) showed excellent therapeutic effects in the collagen-induced arthritis mouse model (Coppieters et al., 2006). Another study evaluating the therapeutic potential of bivalent TNFα nanobodies in a mouse model of chronic colitis impressively demonstrated the versatility of the nanobody technology: genetically engineered *Lactococcus lactis* secreting MT1-MT1 bivalent anti-TNFα nanobodies profoundly reduced gut inflammation when daily administered by gavage (Vandenbroucke et al., 2010). Apart from neutralizing cytokines nanobodies can be used to “guide” cytokines to their desired target cells. In a proof-of-concept study Garcin et al. (2014) demonstrated that the toxicity of type I interferons, when applied *in vivo*, could be markedly reduced by genetically engineering fusion proteins of mutated IFNα2 with lower receptor affinities and nanobodies targeting programmed cell death 1 ligand 2 (PD-L2; Garcin et al., 2014). When injected into mice these fusion proteins preferentially induced IFNα2-mediated STAT1 phosphorylation in PD-L2 expressing cells in peritoneum and spleen, illustrating that nanobodies are valuable tools for “activity-by-targeting” based therapeutic approaches. For the treatment of acute brain injury, the nanobody-based neutralization of proinflammatory cytokines such as TNFα could be a promising approach to minimize inflammation-related further loss of brain tissue. Conversely, it is conceivable that nanobodies could be used to guide modified anti-inflammatory cytokines such as interleukin-10 to sites of brain inflammation to suppress inflammatory responses *in situ*.

### Nanobodies directed against ecto-enzymes

The first cell surface resident ecto-enzyme targeted by nanobodies was murine ADP-ribosyltransferase C2 (ARTC2; Koch-Nolte et al., 2007; Menzel et al., 2013). ARTC2 is expressed on the cell surface of T cells and covalently attaches the ADP-ribose group of its substrate nicotinamide adenin dinucleotide (NAD) to arginine residues of several cell surface proteins. One well-characterized target of ARTC2 is the ATP-gated P2X7 ion channel. ADP-ribosylation of P2X7 on T cells induces channel opening and influx of calcium ions. Prolonged activation by ADP-ribosylation causes shedding of cell surface proteins such as CD62L and CD27, externalization of phosphatidylserin und ultimately cell death (Seman et al., 2003). Analyses of T cell subpopulations revealed different sensitivities to NAD-mediated cell death, with regulatory T cells (Tregs) and natural killer T cells (NKT cells) being highly susceptible (Hubert et al., 2010; Rissiek et al., 2014b). Antagonizing ARTC2 with nanobody s+16a prevents ADP-ribosylation of P2X7 *in vitro* and *in vivo*. In a proof-of-principle study Scheuplein et al. showed that injection of s+16a as Fc-fusion protein restores an otherwise naturally NAD-depleted NKT cell population in diabetogenic NOD-CD38ko mice (Scheuplein et al., 2010). When activated *in vivo* by injection of α-galactosylceramide, s+16a-restored NKT cells were capable of inhibiting the development of type 1 diabetes. A further study showed that injection of s+16a prevented ARTC2/P2X7 mediated cell death of highly susceptible Tregs and NKT cells during *in vitro* assays and adoptive transfer experiments, revealing the potential of s+16a as valuable tool for research and as potential therapeutic agent (Rissiek et al., 2014a). It has been shown that P2X7 activation is detrimental for the outcome of ischemic stroke (Arbeloa et al., 2012). Further, genetic deletion of the NAD-degrading ecto-enzyme CD38 in mice exacerbates ischemic damage (Choe et al., 2011), which might provide ARTC2 with an increased access to its substrate NAD. Therefore, s+16a could be used to clarify the role of the ARTC2/P2X7 axis during acute brain damage.

## Modifying nanobodies to cross the blood-brain barrier

**Figure 2:**
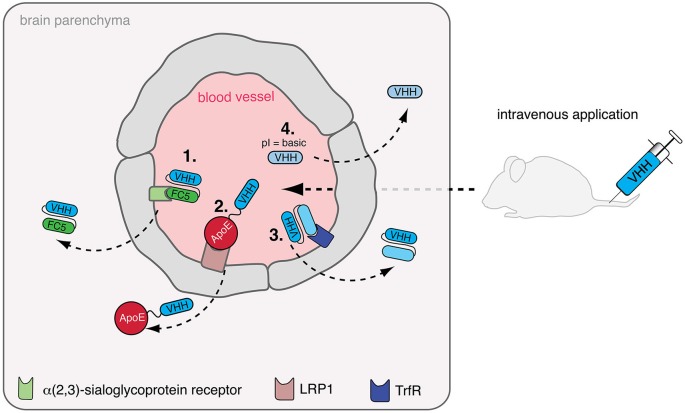
**Delivery of nanobodies to the brain**. The blood-brain-barrier (BBB) hampers the delivery of intravenously injected nanobodies (VHH) to the brain. To overcome this, diverse strategies are being developed: **(1)** Nanobody FC5, binding to a putative α(2,3)-sialoglycoprotein receptor, can potentially be used as shuttling-nanobody to deliver other therapeutic proteins e.g., nanobodies to the brain. **(2)** Apolipoprotein E (ApoE) binds to low density lipoprotein receptor-related protein 1 (LRP1) inducing transcytosis, which can be exploited as shuttle for therapeutic nanobodies. **(3)** In a similare fashion, other receptors triggering transcytosis across the BBB such as the transferrin receptor (TrfR) could be targeted for the transfer of therapeutic nanobodies. **(4)** Finally, shifting the isoelectric point (pI) of therapeutic nanobodies to a basic level facilitates crossing of the BBB by these nanobodies.

The therapeutic application of nanobodies has been tested in diverse mouse models of inflammation. However, therapeutic applications of biologics in neuroinflammatory diseases face an important biological barrier. The obstacle for effective delivery of therapeutic drugs, especially antibodies, is the blood brain barrier, which is only permeable for lipophilic molecules of up to 400 kDa of size (Pardridge, 2012). The delivery of conventional antibodies to the brain is especially tedious because of Fc-receptor mediated efflux to the blood (Cooper et al., 2013). Therefore, nanobodies lacking an Fc-part represent a promising alternative to brain targeting monoclonal antibodies. Indeed, several groups have tested different strategies to deploy nanobodies as brain-drug deliverers or as bonafide brain-targeting drugs (Figure 2).

Muruganadam et al. described in 2002 the selection of a nanobody (FC5) that transmigrates across human blood-brain-barrier endothelium *in vitro* (Muruganandam et al., 2002). Later, the same group suggested that FC5 binds a putative α(2,3)-sialoglycoprotein receptor and is transcytosed via clathrin vesicles (Abulrob et al., 2005). In a therapeutic experimental setup using the Hargreaves model of inflammatory pain, it was shown that FC5 conjugated with opioid peptide Dal could be deployed as drug delivery shuttle *in vivo* to induce a significant analgesic response in contrast to unconjugated Dal peptide (Farrington et al., 2014). Other approaches utilize receptor-mediated transcytosis for brain targeting. A recently published study showed that a fusionprotein of a peptide derived from apolipoprotein E (ApoE) and a model therapeutic protein (α-L-iduronidase) could be transferred to the brain via binding to the LDL receptor (LRP) expressed on cells of the blood-brain barrier (Wang et al., 2013). Furthermore, the transferrin receptor and the insulin receptor have also been exploited for receptor-mediated transcytosis of small molecule drugs and therapeutic proteins (Boado et al., 2012; Xiao and Gan, 2013). These studies indicate that nanobodies binding these receptors and triggering transcytosis could be a promising alternative to ligand-based delivery of drugs to the brain.

A study by Pierre Lafaye’s group (Li et al., 2012) reported that nanobodies with a high isoelectric point (pI) spontaneously cross the blood brain barrier. Such nanobodies not only gained access to the brain but were even found to penetrate cells and bind to intracellular proteins. In a mouse study, the nanobody E9 (pI = 9.4) directed against glial fibrillary acidic protein (GFAP) crossed the BBB after injection via the tail vein and was able to bind to intracellularly expressed GFAP in astrocytes. Conjugation of fluorescent proteins to nanobody E9 (generating a “fluobody”) allowed *in vivo* labeling of astrocytes, however, only if the basic pI was preserved. One possible limitation of this approach is that fairly large amounts (2 mg) of nanobody had to be injected to obtain the desired effect. However, combining this approach—adjusting the pI to a basic level—with other approaches could possibly show beneficial effects. Indeed, the FC5 nanobody described above also has a basic pI (9.2) which might contribute to or facility transcytosis into the brain parenchyma (Farrington and Sisk, 2013). Further studies are needed to determine whether therapeutic nanobodies against inflammatory target proteins can be shuttled to the brain, e.g., by fusion to FC5 and by adjusting their pI with the aim of treating neuroinflammatory disease.

## Implication of nanobodies to treat acute brain inflammation

After acute brain damage, e.g., ischemic stroke or trauma, the release of danger associated molecular pattern (DAMPs) from necrotic cells activates resident microglia, leading to the production of proinflammatory mediators such as cytokines and chemokines attracting other immune cells (Amantea et al., 2009; Iadecola and Anrather, 2011). Preventing local inflammation could be a means to prevent further loss of brain tissue. High mobility group box 1 (HMGB1) and nucleotide DAMPs such as adenosinetriphosphate (ATP) are released during cerebral ischemia (Magnus et al., 2012). Antibody-mediated neutralization of HMGB1 or antagonism of its receptor (receptor for advanced glycation end products, RAGE) markedly reduced the infarct size in a mouse ischemia/reperfusion model middle cerebral artery occlusion (MCAO; Muhammad et al., 2008). The ATP receptor P2X7, which mediates inflammasome formation and cell death (Bartlett et al., 2014), was antagonized with small molecule inhibitors in a mouse model of transient focal ischemia, again resulting in a reduction of infarct size (Arbeloa et al., 2012). Both pathways, HMGB1/RAGE and ATP/P2X7, display promising targets for nanobody-mediated antagonism. However, release of DAMPs occurs shortly after the ischemic insult and before the breakdown of the BBB (Muhammad et al., 2008; Cisneros-Mejorado et al., 2014), requiring the generation of nanobodies that are able to cross the BBB applying the strategies described above. Currently, nanobodies directed against the P2X7 receptor are under development (Laeremans et al., 2010).

An approach to control ischemia-related brain inflammation is to prevent migration of immune cells to the penumbra of ischemic lesions. Two independent studies demonstrated that blockade of the CXCL12/CXCR4 axis improved the functional outcome after stroke by attenuating post-ischemic inflammation (Huang et al., 2013; Ruscher et al., 2013). CXCL12-CXCR4 blockade was conducted using the small molecule inhibitor AMD3100. Since nanobodies against CXCL12 and CXCR4 have been generated (Jähnichen et al., 2010; Maussang et al., 2013), it may be worthwhile to evaluate their potential in ischemia/reperfusion animal models. To restrict the blockade of CXCR4 to infiltrating proinflammatory cells one could apply the “activity-by-targeting” strategy described above for IFNα2-guiding nanobodies. This could be useful in order to allow the CXCR4-dependend migration of cells important for brain recovery after stroke such as mesenchymal stem cells (Tsai et al., 2011). Furthermore, blockade of other chemokine receptors such as CXCR1 and CXCR2 by small molecule inhibitor Reparixin also reduces infiltration of proinflammatory neutrophiles and improves the motoric recovery after stroke (Sousa et al., 2013). Therefore, generation and application of nanobodies directed against CXCR1 and CXCR2 may represent one further strategy to ameliorate consequences of cerebral ischemia and beyond.

## Conclusions

Nanobodies have been shown to be versatile and efficient biologicals suitable for therapy of inflammatory diseases. Due to their unique structure, nanobodies have the potential to modulate the function of cell surface and secreted proteins in an agonistic or antagonistic fashion. They can be genetically engineered to extend their half-life *in vivo* and, shown in a proof-of-concept study, to serve as shuttles for the delivery of therapeutic agents across the BBB. Future studies will have to show whether this strategy could also be applied to deliver therapeutic nanobodies to the brain to ameliorate the consequences of neuroinflammation.

## Conflict of interest statement

The authors declare that the research was conducted in the absence of any commercial or financial relationships that could be construed as a potential conflict of interest.
